# Spontaneous regression of associated aneurysms after management of arteriovenous malformation. A systematic review

**DOI:** 10.1007/s00701-025-06721-2

**Published:** 2026-01-22

**Authors:** Valentina Corpus Gutiérrez, Mariana Angarita Avendaño, Paula Andrea Beltrán Guevara, Felipe Ramirez-Velandia, Laura Bejarano Mora, Juan Carlos Puentes Vargas

**Affiliations:** 1 Neurosurgery Research Group, Pontificia Universidad Javeriana, Bogotá, DC. Colombia; 2 Department of Neurosurgery, Pontificia Universidad Javeriana, Hospital Universitario San Ignacio, Bogotá, Colombia

**Keywords:** Aneurysms, Arteriovenous malformations, Spontaneous regression, Radiosurgery, Endovascular

## Abstract

**Purpose:**

Assess through a systematic review the probability of spontaneous regression of aneurysms after receiving surgical, endovascular, or radiosurgical treatment of AVMs in patients with AVM-associated aneurysms.

**Methods:**

A systematic literature review was performed in May 2025 using PubMed, Embase, and Scopus. The PRISMA flowchart for evidence screening and selection was used. Eligible studies included patients over 18 years of age with intracranial AVMs and associated FRAs, and reported spontaneous aneurysm regression following AVM treatment. Studies were excluded if they had fewer than 10 patients, non-intracranial lesions, involved only conservative management, or were not published in English or Spanish. Data on demographics, clinical presentation, interventions, and outcomes were extracted. The risk of bias was assessed using the ROBINS-I tool, and the certainty of the evidence was evaluated using the GRADE framework.

**Results:**

Out of 264 screened studies, 10 met the inclusion criteria, involving a total of 428 patients. Most studies were retrospective cohorts with a moderate risk of bias. Patient ages ranged from 31 to 58 years, with hemorrhagic presentation in over 50%. AVMs were mostly Spetzler-Martin grades I–III and supratentorial. Proximal and distal aneurysms were more common than intranidal types. Regression rates of aneurysms ranged from 3 to 23%, with no consistent correlation to AVM complete obliteration rates, aneurysm type, or location. Spontaneous aneurysm regression is rare, with a pooled rate of 11% across studies. Despite moderate heterogeneity, sensitivity analyses confirmed the robustness of results. Funnel plots suggested possible publication bias, indicating the true rate may be even lower. Clinically, regression should be considered exceptional, reinforcing the need for vigilant follow-up and individualized treatment.

**Conclusion:**

Spontaneous aneurysm regression after AVM treatment is a recognized but variable and low-frequency phenomenon, with the highest observed probability being 23%. Further studies are needed to guide treatment strategies in these complex cases.

## Introduction

An arteriovenous malformation (AVM) is one of the four cerebrovascular malformations (AVMs, venous angioma, cavernous malformation, and capillary telangiectasia). It consists of a tangle of dysplastic vessels comprising a nidus fed by arteries and drained by veins, but without intervening capillaries [12]. As mentioned, these lesions exhibit three typical features: feeding arteries, draining veins, and a dysplastic vascular nidus, which is composed of a tangle of abnormal vessels that acts as a shunt between the arterial and venous systems [6]. The lack of a capillary bed results in a high-flow, low-resistance conduit that shunts blood from the arterial to the venous system. This chronic high flow shunt produces dilation of the feeding arteries and dilation and thickening of the draining veins. Regarding epidemiology, one brain AVM is found in every 2.000 MRI scans, which adds up to a prevalence of 50 cases per 100.000 [12].

AVMs can be associated with other vascular alterations, such as aneurysms; the rate of this association varies greatly in the literature (2.7–52%) [13]. The pathophysiological explanation of this association is still a subject of study; the evidence supports that it is believed to be secondary to a high-flow vasculopathy, since there is an increased blood flow through the arterial feeders of the AVMs, and this can contribute to the development of aneurysms [1].

Aneurysms associated with AVMs can be classified into two categories: flow-related aneurysms (FRA) and non-flow-related aneurysms (NFRA). Those related to flow constitute 85% of aneurysms associated with AVM. They appear in arteries that supply the AVM and can be proximal, if they are found in the arteries of the circle of Willis or proximal to a primary bifurcation; or distal, if they are far from the primary bifurcation. Within the FRA, we can also find the intranidal ones, which represent 5.5% of the FRA [6]. Those not related to the flow constitute 15% of the aneurysms associated with AVM, located in remote vessels that have no relationship with the substitution of the MAV [6].

The most frequent clinical presentation of aneurysms associated with AVMs is hemorrhage. The source may be rupture of the aneurysm or the AVM, with the latter slightly more common. However, most of the time, the origin is undetermined [18].

For management, embolization, microsurgery, and radiosurgery have been mainly described. There is controversy regarding the timing of aneurysm treatment relative to AVM treatment, since the complex high-flow hemodynamics of AVM and its relationship to the aneurysm make it difficult to concurrently treat both pathologies in the same setting, even endovascularly [23]. It also depends on the clinical context; in the case of intraventricular hemorrhage, the treatment of the lesion responsible for the hemorrhage should be performed first, regardless of whether it is the aneurysm or the AVM. When the source cannot be determined, the treatment strategy is controversial [17]. When the hemorrhage is due to a ruptured aneurysm, the management of these is a priority, given the higher likelihood of re-rupture [21]. However, for unruptured aneurysms, the consequent regression of aneurysms has been described when the AVM is treated first [8]. The explanation for this is related to the possible pathophysiology of aneurysm development associated with blood flow. It is postulated that obliteration of the AVM will decrease blood flow through the feeder vessels, thereby decreasing intravascular pressure and reducing the vessel wall stress, allowing for vessel remodeling and leading to aneurysm resolution [8, 9, 21]. There are other studies that demonstrated this aneurysmal regression after surgical resection or embolization of the AVM [10, 14]. However, there are additional factors that must be taken into account, which can also lead to the spontaneous regression of an aneurysm, such as hypotension, severe vasospasm, the use of antifibrinolytics, and local damage to the artery wall [3].

Given the findings in the literature regarding the management of FRA associated with AVMs, we aimed to describe, through a systematic review, the probability of spontaneous regression of the aneurysm after surgical obliteration of the AVM.

## Methods

### Literature search

A systematic review of the literature was carried out. The search was performed in May 2025 in the PubMed, Embase, and Scopus databases using the following terms: ("intracranial aneurysm" AND "intracranial arteriovenous malformation") AND ("endovascular procedure" OR "microsurgery" OR "stereotactic radiosurgery") AND ("regression" OR "obliterate" OR "disappearance").

### Study selection criteria

**Fig. 1:**
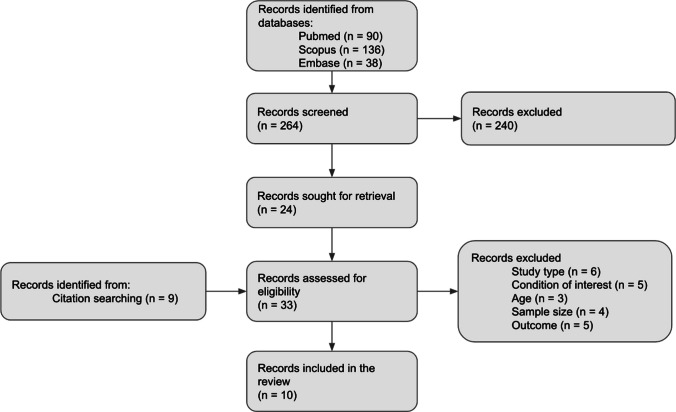
PRISMA flowchart for evidence screening and selection

The PRISMA flowchart for evidence screening and selection was used (Fig. 1). The results were initially filtered according to title and abstract by all members of the research team using the Rayyan platform and considering the following inclusion criteria: patients over 18 years of age, with flow related aneurysms (FRA) associated with arteriovenous malformation (AVM) of both hemorrhagic and non-hemorrhagic presentation and studies since 1925 that describe the surgical management of FRA associated with AVM and evaluate the rate of spontaneous obliteration of the aneurysm following the treatment of the AVM. Studies with fewer than 10 patients, those involving intracranial lesions, or those that only included patients with aneurysms not associated with AVM, AVM not associated with aneurysms, aneurysms not related to flow, or other associated lesions were excluded. Additionally, studies that only described conservative management and were written in languages other than Spanish or English were excluded. Once the articles to be included in the review are defined, a “snowball” search was carried out for references that may be useful.

### Data extraction

Study type, sample size, patient demographics, hemorrhagic presentation, vascular injury, the intervention performed, and the rates of spontaneous regression of the aneurysm after surgical obliteration of the AVM were extracted from the included studies.

### Data syntheses and analysis

To synthesize individual study results, we performed a meta-analysis of proportions. Forest plots were generated to visually display the regression rates of AVM-associated aneurysms across studies, along with 95% confidence intervals. Anticipating clinical and methodological variability, we employed a random-effects model to obtain a pooled estimate and address between-study heterogeneity. Funnel plots were also built to qualitatively assess potential publication bias or small-study effects.

### Methodological quality

The methodological quality and risk of bias of the included articles were assessed using the ROBINS-I tool. Among the studies included, 1 was classified as low and 9 as moderate (Table 1). To determine the certainty of the evidence, we utilized the Grading of Recommendations, Assessment, Development, and Evaluation (GRADE) methodology (Table 2). This method categorizes the certainty of evidence into four levels: very low, low, moderate, or high. It evaluates four factors: risk of bias, inconsistency, direct evidence, and imprecision. The evidence certainty is then summarized in a table for each outcome using the GRADEpro virtual platform.

**Table 1:**
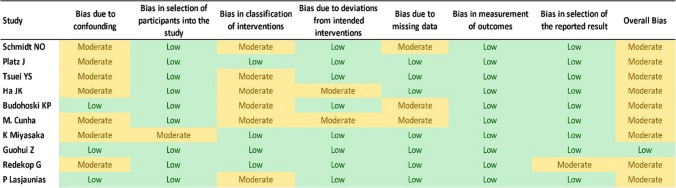
ROBINS-I tool

**Table 2:** Grading of recommendations, assessment, development, and evaluation (GRADE) for assessing the certainty of the evidence

Outcome	No. of Studies	Sample Size	Effect Estimate	Risk of Blas	Inconsistency	Indirectness	Imprecision	Other Considerations	Certainty (GRADE)	Importance	Comments
Spontaneous regression rate of aneurysms after AVM management	10	**428**	3%−23%	Moderate	Moderate to high heterogeneity	None	Not assessed	None	Low	Critical	Variability among studies, no confidence intervals reported

## Results

### Study selection

264 studies were screened by title and abstract, from which 24 were included in the full-text review. From those, 10 studies were finally chosen for data extraction (Fig. 1). Among these 10 studies, the outcomes of 428 patients with AVM-associated feeder aneurysms were selected to show the rates of spontaneous regression of the aneurysm.

### Study characteristics

Seven of the included studies were retrospective cohorts, one was a prospective cohort series, one was a case series, and one was a cross-sectional study (Table 3). Among them, one had a low risk of bias and nine had a moderate risk of bias (Table 1).

**Table 3:** Demographic and clinical characteristics

Study	Study Type	Mean Follow-up	Sample size	Mean Age	Females	Hemorrhagic presentation
Schimdt NO, 2011 [20]	Retrospective cohort	6 months	17 p	31 y	44%	65%
Cunha MJ, 1992 [5]	Retrospective cohort	55 months	39 p	38 y	33%	62%
Tsuei YS, 2019 [22]	Cohort	58 months	72 p	43 y	40%	43%
Redekop G, 1998 [19]	Retrospective cohort	3.8 years	33 p	37 y	46%	38%
Zhu G, 2015 [24]	Retrospective cohort	62 months	42 p	32 y	31%	81%
Platz J, 2014 [16]	Retrospective cohort	38 months	59 p	47 y	52%	61%
Ha JK, 2009 [7]	Retrospective cohort	48 months	21 p	46 y	24%	81%
Budohoski KP, 2021 [2]	Retrospective cohort	35 months	32 p	58 y	69%	-
Miyasaka K, 1982 [15]	Cross—sectional	-	22 p	41 y	56%	27%
Lasjaunias P, 1998 [11]	Case series	-	27 p	38 y	33%	57%

*p* Patients, *y* Years

### Demographic characteristics, clinical presentation, and follow-up

In total, there were 364 patients between 31 and 58 years. The number of women varied, with percentages ranging from 24 to 69%.

The hemorrhagic presentation was an important feature. It was observed that 27%—81% of patients presented intracranial bleeding. In almost all studies, the percentage of hemorrhagic presentation was greater than 50%, which can be explained by the greater risk of patients who have aneurysms associated with AVM.

The mean follow-up varied between 6 months and 5 years (Table 3).

### Arteriovenous malformation characteristics, aneurysm characteristics and follow-up

In all studies, there were more AVMs classified as Spetzler-Martin (SM) I–III. The location was mostly supratentorial, except for studies such as Zhu et al. (2015) and Schmidt et al. (2011), which included patients with infratentorial AVMs in their study population (Table 4).

**Table 4:** Characteristics of the injuries, management and rate of AA spontaneous regression

Study	SM I—II %	SM III %	SM IV-V %	AVM < 3 cm	AVM 3–6 cm	AVM > 6 cm	Infratentorial(i) or deep (d) location	IA # (%)	DA # (%)	PA # (%)	RA # (%)	Intervention	Injury managed first	% AVM oblit	AA regression % (No.)
SchimdtNO, 2011 [20]	61	26	13	-	-	-	100% (i)	4 (22)	14 (78)		-	EVTº✞, Sº✞, RSº	AVM	63	22 (4/18)
Cunha MJ, 1992 [5]	-	-	-	21%	28%	51%	20.5% (d)	18 (28)		45 (70)	1 (2)	EVTº✞, Sº✞	AVM	-	9 (6/64)
Tsuei YS, 2019 [22]	-	-	-	-	-	-	-	0	39 (35)	72 (65)	-	RS°	AVM	72	23 (26/111)
Redekop G, 1998 [19]	-	-	-	-	-	-	-	-	10 (17)	48 (83)	-	Sº, EVTº	AVM	52	12 (7/58)
Zhu G, 2015 [24]	50	40	10	71%	29%		100% (i)	14 (22)	49 (78)		-	EVTº✞, RSº, Sº	AA/AVM	75	3 (2/61)
Platz J, 2014 [16]	46	37	17	-	-	-	37.3% (i)	7 (8)	15 (16)	61 (66)	9 (10)	Sº✞, EVTº✞, RSº	Simultaneous	50	3 (1/38)
Ha JK, 2009 [7]	57	29	14	-	-	-	19% (i) 23.8% (d)	13 (48)	-	11 (41)	3 (11)	RSº, S✞, EVT✞	AVM	76	15 (4/27)
Budohoski KP, 2009 [2]	78	13	9	-	-	-	40.6% (i)	10 (20)	20 (42)	11 (23)	4 (9)	Sº✞, RSº, EVT✞	AVM	-	17 (8/48)
Miyasaka K, 1982 [15]	-	-	-	23%	51%	26%	0 (i)	43				EVTº, Sº	AVM	-	7 (3/43)
Lasjaunias P, 1998 [11]	-	-	-	-	-	-	0% (i) 70% (d)	7 (19)		21 (57)	9 (24)	EVTº, Sº	AVM	-	8 (3/37)

*SM* Spetzler-Martin Grading Scale, *AVM* arteriovenous malformation, *DVD* deep venous drainage, *IA* intranidal aneurysms, *DA* distal aneurysms, *PA* proximal aneurysms, *RA* remote aneurysms, *RS* radiosurgery, *EVT* endovascular therapy, *S* surgery, *obilt* Percentage of AVMs with complete obliteration, *m* months, *AA* Associated aneurysm

º Actual management of the AVM

✞ Actual management of the aneurysm

* Prior management of the aneurysm

‘’ Prior management of the AVM

*(i)* Cerebellum or brainstem

*(d)* Thalamus, basal ganglia, hypothalamus, ventricles or corpus callosum

Regarding the aneurysms associated with the AVM, the most common were the proximal and distal ones, with the intranidal ones being the rarest. Additionally, many studies included non-flow-related (or remote) aneurysms in their analysis (Table 4).

The mean size was 4.1—5 mm, and many patients were found with more than 1 aneurysm (30–50%).

### Regression rates

Aneurysm regression rates after AVM management were 3–23%.

No correlation was found between spontaneous aneurysm regression and AVM complete obliteration rate (that was 50–76%) or the location of the AVM (Table 4).

### Results of syntheses and heterogeneity among study results

**Fig. 2:**
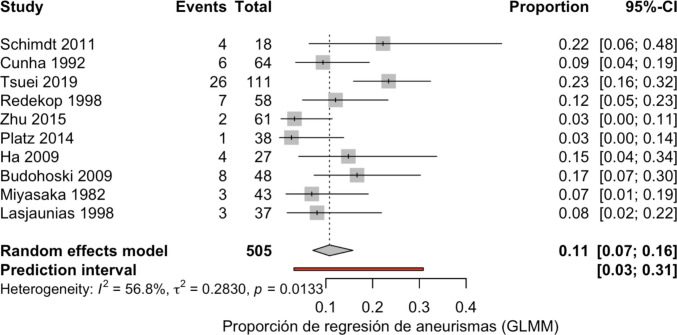
Meta-analysis

A random-effects meta-analysis demonstrated an overall aneurysm regression rate of 11% (95% CI: 7%–16%) following AVM treatment (Fig. 2). The prediction interval ranged from 3 to 31%, indicating substantial variability in effect sizes across studies. Regression rates reported by individual studies varied widely, from as low as 3% to as high as 23%. Statistical heterogeneity was moderate (I^2^ = 56.8%, τ^2^ = 0.283, *p* = 0.013), suggesting that more than half of the variability was attributable to differences in study design, patient populations, aneurysm characteristics, and treatment strategies rather than chance alone. These findings indicate that although spontaneous regression of AVM-associated aneurysms does occur, it is relatively uncommon and highly inconsistent across published cohorts.

Only three studies stratified regression rates according to aneurysm location (proximal vs. distal). In these reports, regression appeared more frequent in distal aneurysms compared to proximal aneurysms in two of the three studies (Table 5). However, due to the limited number of studies reporting this variable, no formal subgroup or meta-regression analysis could be performed. These findings should therefore be interpreted with caution and considered hypothesis-generating. In addition, other factors such as treatment modality, aneurysm size, AVM subtype, and duration of follow-up may also influence the probability of regression; nevertheless, the available data across studies were insufficient to allow formal stratification or quantitative analysis of these variables.

**Table 5:** Regression according to aneurysms proximity

	Distal aneurysms	Proximal aneurysms	*p* =
Tsuei YS, 2019 [22]	55%	6%	< 0.001
Redekop G, 1998 [19]	60%	2%	-
Budohoski KP, 2009 [2]	43%	50%	0.04—0,34

*AA* Associated aneurysm, *AVM* arteriovenous malformation, *SM* Spetzler-Martin Grading Scale, *DA* distal aneurysms, *PA* proximal, aneurysms, *RS* radiosurgery, *EVT* endovascular therapy, *S* surgery

### Sensitivity analysis

**Fig. 3:**
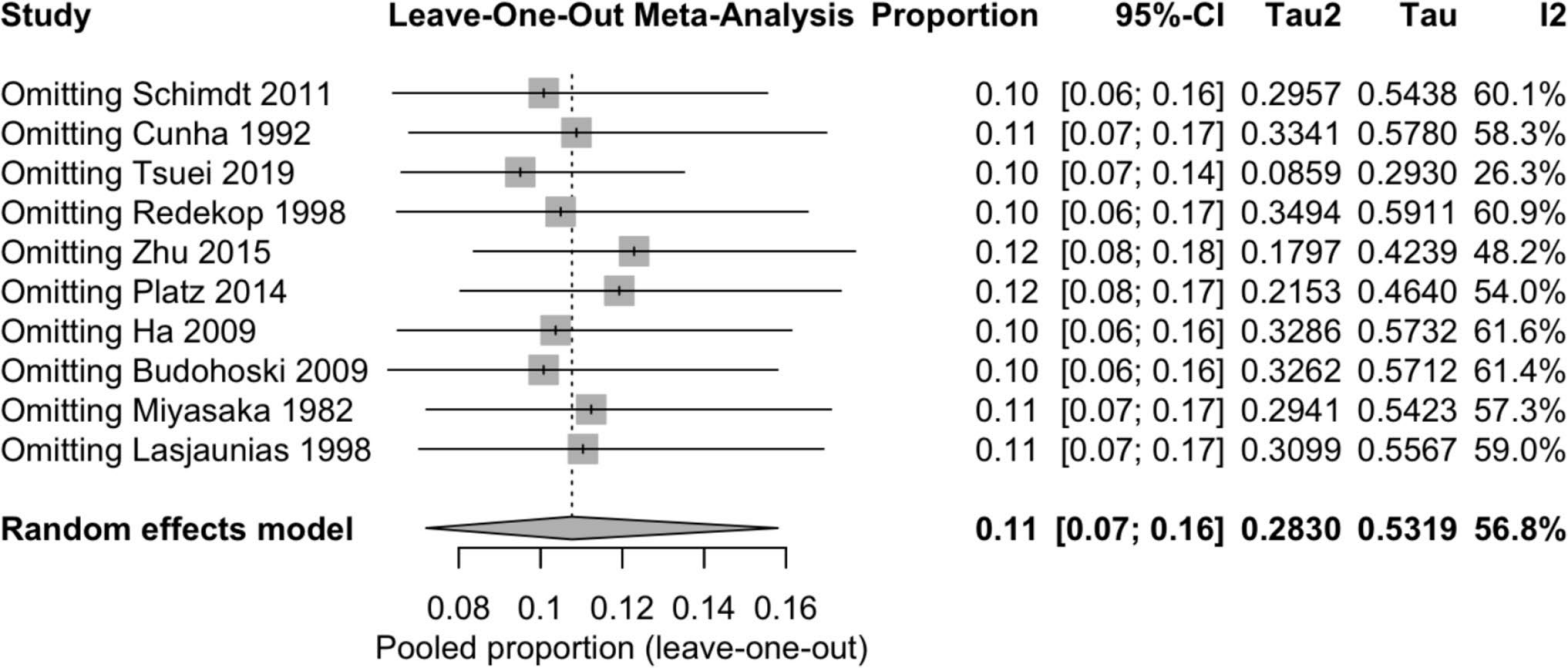
Sensitivity analysis

**Fig. 4:**
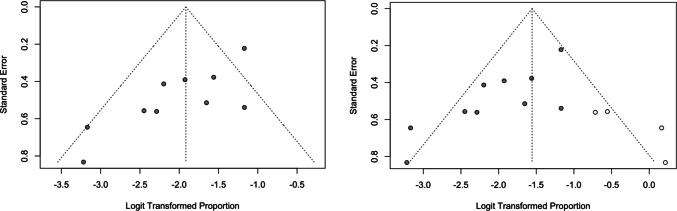
Funnel plot

Sensitivity analyses revealed that excluding individual studies (leave-one-out) did not materially affect the summary estimate, with pooled proportions remaining stable (0.10–0.12), thereby confirming the robustness of the findings (Fig. 3). Influence diagnostics indicated that no single study exerted a disproportionate effect on the overall results, although studies with larger sample sizes (e.g., Tsuei 2019) contributed more weight to the model. The Baujat plot suggested that heterogeneity was not driven by a single outlier but rather distributed across multiple studies. Funnel plot inspection revealed relative symmetry; however, trim-and-fill procedures suggested the potential presence of small-study or publication bias, which could have led to a slight overestimation of the pooled effect (Fig. 4).

## Discussion

The present systematic review investigated the possibility of spontaneous regression of FRA following treatment for AVMs. The pathophysiological hypothesis states that flow-related aneurysms develop as a consequence of hemodynamic stress imposed by the AVM on the feeding arteries. Once the AVM is obliterated, the abnormal high-flow shunt is disrupted, potentially reducing arterial wall stress and promoting vessel remodeling. This is especially relevant in cases of distal and intranidal aneurysms, which are more directly influenced by AVM-related hemodynamics. In the case of distal aneurysms, this happens because most distal feeders of the AVM provide blood only to the AVM, so when total obliteration of the AVM occurs, a total reduction in the caliber of the distal feeding artery is generated, and therefore, total or partial regression of the aneurysm. On the contrary, the main feeding arteries of the AVM supply blood to the AVM but also to normal brain tissue, so the occlusion of the AVM, although it reduces the hemodynamic stress of these parental vessels, the flow of these vessels continues feeding the aneurysm within them, reducing the frequency of spontaneous regression of proximal flow-related aneurysms.

This was demonstrated in our included studies. Within these, we evidenced that the highest regression rate was presented in Tsuei YS et al. [22], in which both partial (23%) and complete (23%) regression were documented; in both cases, distinctively, this regression occurred predominantly in distal flow-related aneurysms, rather than in proximal flow-related aneurysms. Even so, the percentage of aneurysms that maintained a stable size was higher (54%). Similarly, in the study by Budohoski et al. [2], a statistically significant regression was observed in distal aneurysms compared to proximal ones, with a 51% and 58% reduction in height and width, respectively (*P* = 0.046 and *P* = 0.028).

Regarding the behavior of AVMs according to their location, it has been described that aneurysms located in the feeding arteries of posterior fossa AVMs present a higher risk of hemorrhage, Schmidt NO et al. [20] suggest that in these cases, the priority should always be the management of the aneurysm, rather than the obliteration of the AVM. It is striking that they conclude this despite the fact that all patients in whom only the AVM management was performed presented spontaneous regression of the aneurysm at 6 months.

When comparing complete regression to partial shrinkage, studies have reported a higher frequency of shrinkage than complete resolution. For example, in the study by Budohoski et al., 35% of aneurysms showed shrinkage, while only 21% demonstrated complete regression—all of which were distal aneurysms. This may be explained by the fact that most AVMs do not coexist with flow-related aneurysms, suggesting the presence of an underlying deficiency in the vessel wall. Consequently, complete resolution of the aneurysm may not always be achievable [2].

Although some studies, as mentioned, demonstrated spontaneous regression of the aneurysm after AVM management, this finding was not consistently observed in the remaining studies, as the percentages of regression varied from 3 to 23%. Therefore, as a conclusion of the systematic review, we can conclude that spontaneous regression of aneurysms after AVM management is not consistently observed in clinical practice.

Our meta-analysis suggests that spontaneous aneurysm regression is an uncommon phenomenon, with a pooled proportion of approximately 11% across the available studies. Although the estimates were consistent across different statistical models, the presence of moderate heterogeneity (I^2^ ≈ 55–64%) highlights variability between studies, likely reflecting differences in patient populations, aneurysm characteristics, imaging follow-up protocols, and study designs. Importantly, sensitivity analyses confirmed the stability of the results, as no single study exerted undue influence on the pooled estimate. This robustness strengthens the reliability of the observed low frequency of aneurysm regression.

Additionally, during the same time this study was conducted, another systematic review and meta-analysis [4] was conducted to evaluate the rate of postoperative hemorrhage (PH) associated with aneurysms in patients with AVMs, comparing rates when the AVM is treated first versus when the aneurysm is treated first. It was demonstrated that the hemorrhage rate when the AVM was treated first was 8.42%, compared to 3.37% when the aneurysm was treated first. This finding allows us to conclude that the rate of postoperative hemorrhage is higher when the AVM is treated first and lower when the aneurysm is treated first. Aiming to comparatively evaluate the probability of aneurysm regression when the AVM is treated first, we conducted this parallel study, in which we concluded that the probability of such regression is neither significant nor consistent across studies. With these two findings, we can conclude that the management of the aneurysm should be considered first to avoid the high risk of PH. This should always be done by analyzing each case, its risks, and the disadvantages of each procedure for each patient separately.

Some limitations were identified in this study. First, all studies included were observational, and nine had a moderate risk of bias, mainly due to confounding and unclear intervention classification. The heterogeneity in patient populations, follow-up times, and aneurysm characteristics also limits the generalizability of the results. Additionally, some studies included remote or non-flow-related aneurysms in their analysis, which could have influenced the observed regression rates.

Additionally, the funnel plot and trim-and-fill analyses suggest a potential publication bias, where small negative studies might remain unpublished. This could imply that the true incidence of regression is even lower than our estimate. Clinically, these findings underscore that while spontaneous regression is possible, it should be regarded as an exceptional event rather than a predictable outcome. Careful monitoring and individualized treatment planning remain essential, as relying on spontaneous regression would carry significant risks for most patients.

Further prospective studies with standardized classification of aneurysm types and robust clinical endpoints are needed to clarify the safety and efficacy of treating AVMs first in this patient population. Until such data are available, treatment decisions should be individualized, balancing the hemorrhagic risk of each lesion and considering the potential for aneurysm regression after AVM management.

## Conclusion

This review suggests that, although the spontaneous regression of aneurysms following AVM treatment has a plausible biological basis, it is not consistently observed in clinical practice. The highest reported probability of regression was 23% when the AVM was treated first.

However, the heterogeneity of the included studies—in terms of design, lesion characteristics, and treatment strategies—limits the ability to draw definitive conclusions. Further prospective studies with standardized methodologies and lower risk of bias are needed to determine the optimal management approach for patients with AVM-associated aneurysms.

## Data Availability

No datasets were generated or analysed during the current study.
